# Open science practices among authors published in complementary, alternative, and integrative medicine journals: An international, cross-sectional survey: Erratum

**DOI:** 10.1097/MD.0000000000043469

**Published:** 2025-07-04

**Authors:** Jeremy Y. Ng, Brenda X. Lin, Liliane Kreuder, Holger Cramer, David Moher

In the article “Open science practices among authors published in complementary, alternative, and integrative medicine journals: An international, cross-sectional survey”^1^, which appears in Volume 103, Issue 44 of *Medicine, was originally published with a CCBY-NC version of the Open Access license. It should have been published with the CC-BY version of the Open Access license. This has now been corrected in the online version.*
